# Facilitative Effects of Transcranial Direct Current Stimulation on Semantic Memory Examined by Text-Mining Analysis in Patients With Schizophrenia

**DOI:** 10.3389/fneur.2021.583027

**Published:** 2021-02-11

**Authors:** Chika Sumiyoshi, Zui Narita, Takuma Inagawa, Yuji Yamada, Kazuki Sueyoshi, Yumi Hasegawa, Aya Shirama, Ryota Hashimoto, Tomiki Sumiyoshi

**Affiliations:** ^1^Faculty of Human Development and Culture, Fukushima University, Fukushima, Japan; ^2^Department of Preventive Intervention for Psychiatric Disorders, National Center of Neurology and Psychiatry, Kodaira, Japan; ^3^Department of Psychiatry and Behavioral Sciences, Stanford University, Palo Alto, CA, United States; ^4^Department of Psychiatry, National Center Hospital, National Center of Neurology and Psychiatry, Kodaira, Japan; ^5^Department of Pathology of Mental Diseases, National Institute of Mental Health, National Center of Neurology and Psychiatry, Kodaira, Japan; ^6^Department of Psychiatry, Graduate School of Medicine, Osaka University, Osaka, Japan

**Keywords:** schizophrenia, tDCS, semantic memory, category fluency, text-mining analysis

## Abstract

**Background:** Beneficial effects of transcranial direct current stimulation (tDCS) are relevant to cognition and functional capacity, in addition to psychiatric symptoms in patients with schizophrenia. However, whether tDCS would improve higher-order cognition, e.g., semantic memory organization, has remained unclear. Recently, text-mining analyses have been shown to reveal semantic memory. The purpose of the current study was to determine whether tDCS would improve semantic memory, as evaluated by text-mining analyses of category fluency data, in patients with schizophrenia.

**Methods:** Twenty-eight patients entered the study. Cognitive assessment including the category fluency task was conducted at baseline (before tDCS treatment) and 1 month after t administration of tDCS (2 mA × 20 min, twice per day) for 5 days, according to our previous study. The category fluency data were also obtained from 335 healthy control subjects. The verbal outputs (i.e., animal names) from the category fluency task were submitted to singular valued decomposition (SVD) analysis. Semantic memory structures were estimated by calculating inter-item cosines (i.e., similarities) among animal names frequently produced in the category fluency task. Data were analyzed longitudinally and cross-sectionally to compare the semantic structure within the patient group (i.e., baseline vs. follow-up) and between groups (patients vs. healthy controls). In the former, semantic associations for frequent items were compared in the form of cosine profiles, while in the latter, the difference in the magnitude of the correlations for inter-item cosines between healthy controls and patients (baseline, follow-up) was examined.

**Results:** Cosine profiles in the patient group became more cluster-based (i.e., pet, carnivores, and herbivores) at follow-up compared to those at baseline, yielding higher cosines within subcategories. The correlational coefficient of inter-item cosines between healthy controls and patients was significantly greater at follow-up compared to baseline; semantic associations in patients approached the normality status after multi-session tDCS.

**Conclusions:** To our knowledge, this is the first study to demonstrate the facilitative effect of tDCS on semantic memory organization in patients with schizophrenia. Text-mining analysis was indicated to effectively evaluate semantic memory structures in patients with psychiatric disorders.

## Introduction

Several domains of cognitive function, specifically, verbal fluency, working memory, and processing speed, are impaired in patients with schizophrenia (1, 2). The cognitive decline compared to healthy adults is in a range of 0.5–2.5 SD (3, 4), hindering functional recovery (5).

Cognitive profiles specific to schizophrenia have been evaluated comprehensively by cognitive batteries, including the Brief Assessment of Cognition in Schizophrenia [BACS; Keefe et al. (6)] and MATRICS Consensus Cognitive Battery [MCCB; Nuechterlein and Green (7)]. Most subtests in these neuropsychological batteries are designed to evaluate executive aspects of cognition (i.e., attention, processing speed, and visual/verbal working memory). Therefore, additional methods are required to assess higher-order cognitive functions, such as semantic memory.

Semantic memory represents a long-term storage of information (8, 9), and semantic structure is defined based on its cohesiveness, i.e., semantic association between items (10). Typically, the semantic structure is represented in the form of clusters, spatial constellations, or networks.

Previous studies have demonstrated aberrant structures of semantic memory in patients with schizophrenia (11–15). Importantly, the disturbance of semantic memory is related with negative symptoms (e.g., alogia) (15) and quality of life (16). These observations indicate the need for the development of effective methods to assess semantic memory in patients with schizophrenia.

Semantic memory is estimated by using data from several cognitive tasks. Specifically, the category fluency task has been used in the study of schizophrenia (11–15). In this task, subjects are instructed to freely recall as many items in a given category (e.g., animal) as possible in a designated time (typically 1 min.). The task is not demanding, and is included in major neurocognitive test batteries, e.g., the MCCB and BACS.

The recent application of text-mining techniques to data from the category fluency task provides objective indices of semantic structures in clinical subjects. For example, network analysis found several parameters, i.e., diameter, average shortest path, and network density, which effectively identify cognitive impairment (17). For the same purpose, latent semantic analysis [LSA; Landauer and Dumais (18)] and singular value decomposition analysis [SVD; Sung et al. (19)] have also been used (19–21). Generally, these methods use a cosine value and vector length to evaluate semantic memory structure (19, 20, 22). The former represents cohesiveness while the latter indicates unusualness of items composing semantic memory. Assuming that disorganization of semantic memory is one of the intermediate cognitive phenotypes of schizophrenia, Nicodemus et al. (20) examined candidate genes related with semantic memory formation by using LSA of category fluency data. They found that average vector length of items was associated with DISC1 in men with schizophrenia. Meanwhile, Sung et al. (19) and Sumiyoshi et al. (21) used SVD analysis, and reported cosine profiles of patients with schizophrenia were deviated from those of healthy controls, revealing unusual structure of semantic memory.

To ameliorate cognitive impairments in schizophrenia, pharmacological, psychosocial, and neuromodulatory approaches have been attempted. Specifically, some types of brain stimulation, particularly non-invasive methods, e.g., transcranial magnetic stimulation and transcranial direct current stimulation (tDCS) have been drawing attention (23, 24). tDCS modulates neural activities in the brain with weak electrical currents (23, 24). The beneficial effects of tDCS are relevant to cognition as well as psychiatric symptoms, functional capacity, and depression in patients with schizophrenia (25, 26).

Although evidence has been accumulated regarding the efficacy of tDCS on cognitive impairment of schizophrenia (26), only a few studies have been conducted to determine whether tDCS would improve higher-order cognition. For example, Vannorsdall et al. (27) reported that tDCS facilitated retrieval of semantically related words in healthy adults. Also, the facilitative effect of tDCS has been found to be more pronounced in category, rather than letter fluency performance (28). These observations suggest that the cognitive enhancement with tDCS is not limited to attention and executive functions, but is also beneficial for a higher level cognitive function, e.g., organization of semantic memory. Thus, it was hypothesized that tDCS would be effective to improve semantic memory structure in patients with schizophrenia.

**Figure 1:**
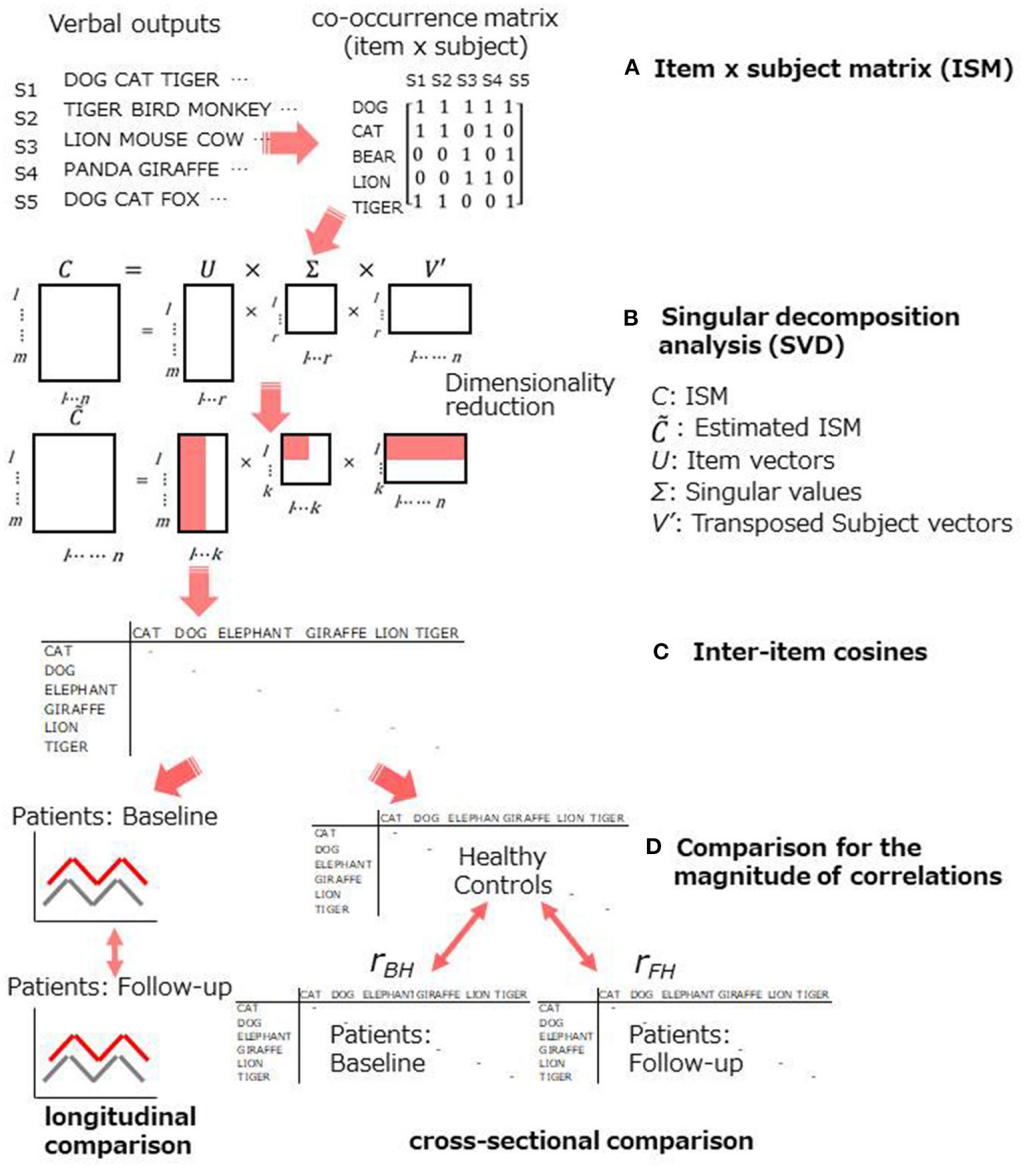
Schematic representation for the statistical procedure. **(A,B)**: an item x subject matrix was produced to submit to SVD analysis, **(C)**: cosine values were used to evaluate the semantic memory structure, **(D)**: improvement was assessed within a group and between groups.

The aim of the current study was to determine whether tDCS would improve semantic structure, as evaluated by text-mining analyses of category fluency data, in patients with schizophrenia. For this purpose, data were analyzed to compare the semantic structure longitudinally (within the patient group: data at baseline vs. those after tDCS administration) and cross-sectionally (between groups: patients vs. healthy controls), as demonstrated in Figure 1D.

## Methods

### Participants

A total of 28 participants were inpatients (*n* = 22) or outpatients (*n* = 6) treated at National Center Hospital, National Center of Neurology and Psychiatry (25). They met DSM-5 criteria for schizophrenia. Patients with alcohol or substance disorder, traumatic brain injury, or epilepsy were excluded. The patients received antipsychotic drugs (25), which were not changed throughout the sessions. Healthy volunteers (*N* = 335) were recruited from the community through local advertisements at Osaka University as participants in a general cognitive assessment (29, 30). They were evaluated using the non-patient version of the Structured Clinical Interview for DSM-IV (SCID) to exclude individuals who had current or past contact with psychiatric services or had received psychiatric medication (31, 32). Data was extracted from our previous study of the effect of tDCS on cognitive function in patients with schizophrenia (25), and from text-mining study using healthy adults (21).

This study was approved by Ethical Committee of National Center of Neurology and Psychiatry, Research Ethics Committee of Fukushima University, and Ethical Committee of Osaka University. The procedures were conducted according to the Declaration of Helsinki and all subjects gave written informed consents.

### Intervention

tDCS was administered according to a method previously reported (33) in line with a previous study of tDCS on cognition in patients with Schizophrenia (34). Participants underwent 10 active tDCS sessions in 5 consecutive days, twice per day. On each day, tDCS intervention was performed approximately at 10 a.m. and 2 p.m. Patients received no additional behavioral treatment or therapeutic adjustment other than tDCS.

Possible adverse effects related to tDCS, including itching, tingling, headache, burning sensation and discomfort, were monitored using semi-structured checklist (35) after each intervention.

A Soterix Medical 1 × 1 Transcranial Direct Current Low-Intensity Stimulator Model 1,300 A was used for the tDCS through two 35 cm^2^ electrodes. We usually soaked 4 ml of saline per side (8 ml into each sponge). For each session, direct current of 2 mA for 20 min was applied. The tDCS montage comprised placement of the anode over the left dorsolateral prefrontal cortex (DLPFC) and the cathode over the right supraorbital area (corresponding to F3 and FP2, according to the International 10–20 electroencephalography system).

### Assessment for Cognition and Psychiatric Symptoms

Cognitive function was assessed at baseline and 1-month after the last tDCS administration using the BACS. Verbal outputs of the category fluency task were obtained from the BACS. Category fluency is a free recall task, asking subjects to produce as many animal names as possible in 1 min. According to the normative method (36), errors (i.e., repetitions, proper nouns, and intrusions [e.g., *APPLE* for an animal cue]) were removed from the analysis. Premorbid intelligence was estimated at baseline using the Japanese version of the Adult Reading Test [JART, Matsuoka et al. (37)]. As for healthy controls, category fluency task and the JART were conducted in a general cognitive assessment (29, 30).

Psychiatric symptoms were assessed at baseline and follow-up using the Positive and Negative Syndrome Scale [PANSS; Kay et al. (38)].

### Statistical Analysis

Demographic variables and category fluency scores were compared between patients and healthy controls using *t*-test. Comparisons between baseline and follow-up in patients were conducted based on our previous report (25). Inequality of variance between the groups was examined using Levene test. Welch method was applied if inequality was significant.

To evaluate the semantic structure, SVD analysis was conducted for verbal outputs of the category fluency task. Figure 1 demonstrates schematic representation of the procedure. First, an item x subject matrix (ISM) was created. Rows of the ISM contained animal items (e.g., *DOG CAT*, etc.), while columns contained subjects, and each cell contained a co-occurrence of items (Figure 1A). Then, SVD analysis was applied to the matrices obtained from patients and healthy controls (Figure 1B). SVD is a general matrix factorization technique based on eigenvalue decomposition [for further information, see Supplementary Materials in Sung et al. (19, 22, 39)]. Each row (i.e., item) is treated as a vector in the space produced by SVD.

A key component of the structure of semantic memory is cosine values in reduced (i.e., higher) dimensions (Figure 1C). A cosine close to 1.0 indicates that two items are highly similar (two words frequently co-occur across subjects).

To assess the improvement on semantic memory structure, cosines between the highly frequent items were contrasted longitudinally and cross-sectionally. In the former, cosine profiles of the 6 most frequent items were produced for patients at baseline and at follow-up and compared (Figure 1D, left). As for the latter, the improvement was evaluated as follows: (1) inter-item cosines were obtained between the 6 most frequent items; (2) Pearson's correlational coefficients for those cosines were calculated between healthy controls and patients at baseline (*r*_BH_) and follow-up (*r*_FH_); (3) The difference in the magnitude of the two correlational coefficients were tested by the Meng's method (40) (Figure 1D, right). The method was employed because the healthy control group was used as a “reference,” and therefore, it was “overlapped” in testing the magnitude of the difference. The significance level was set for *p* < 0.05 with one-tailed (i.e., *r*_*BH*_ < *r*_*FH*_), hypothesizing that the tDCS treatment could improve higher, as well as lower, level of cognition.

R version 3.2.2 (41) and its LSA package (42) were used for conducting SVD analysis and producing inter-item cosines. For testing correlations, R based software cocor (43) was used. Other statistical analyses were conducted by SPSS ver. 22.

## Results

### Demographic and Cognitive Variables

Table 1 presents demographic and clinical variables at baseline and category fluency performance. Inequality of variances was significant only in Estimated premorbid IQ (*F* = 12.22, *p* < 0.001) to which Welch method was applied. Healthy controls were significantly younger, more educated, and showed higher premorbid IQ compared to patients. The former group also produced more words in the category fluency task.

**Table 1:** Characteristics of praticipants^a^.

	**Healthy controls** ***N*** **=** **335**		**Patients** ***N*** **=** **28**				
**Variables**	**M**	**SD**	**M**	**SD**	**x^**2**^/t**	**df**	***p***
M/F	154/181		16/12		1.295	1	0.255
Age (year)	35.8	11.9	40.9	9.8	−2.205	361	0.028
Education (year)	15.2	2.2	13.8	1.7	3.164	361	0.002
Estimated premorbid IQ (JART^b^)	109.3	12.2	99.6	12.0	3.262	29	0.003
Category fluency (Baseline)	20.9	4.5	16.4	5.1	5.071	361	0.000
Category fluency (Follow-up^c^)			16.9	5.5	4.475	361	0.000
Age at onset (year)	–		23.6	6.7			
Duration of illness (year)	–		17.4	9.9			
Neuroleptics (CPZ)	–		889.0	587.2			
PANSS^d^ Positive syndrome	–		15.7	5.7			
PANSS Negative syndrome	–		14.9	8.0			
PANSS General psychopathology	–		32.0	8.1			

a *Demographic variables and PANSS are baseline scores. For the follow-up PANSS scores, see Narita et al. (25) for details*.

b *JART, Japanese Adult Reading Test*.

c *Scores at Baseline and Follow-up were not statistically different (t = 0.56, df = 27 p = 0.58). See Narita et al. (25) for details*.

d *PANSS, the Positive and Negative Syndrome Scale*.

### SVD Analysis

Table 2 presents 20 items most frequently produced by patients and healthy controls. Out of them, 12 items, i.e., BEAR, BIRD, CAT, DOG, ELEPHANT, GIRAFFE, LION, MONKEY, MOUSE, PANDA, RABBIT, TIGER, were chosen for SVD analysis. They commonly appeared at baseline and follow-up, with the frequency more than 10 (Table 2, in bold).

**Table 2:** Frequencies of animal items.

**Rank**	**Healthy controls (*****N*** **=** **335)**		**Patients (*****N*** **=** **28)**			
			**Base**		**Follow-up**	
1	**DOG**	309	**DOG**	24	**CAT**	24
2	**CAT**	305	**LION**	23	**DOG**	24
3	**LION**	250	**CAT**	22	**LION**	23
4	**GIRAFFE**	244	**ELEPHANT**	21	**ELEPHANT**	19
5	**TIGER**	239	**GIRAFFE**	21	**MONKEY**	15
6	**ELEPHANT**	235	**MOUSE**	17	**TIGER**	15
7	**MONKEY**	234	**TIGER**	17	**BIRD**	13
8	HORSE	171	HORSE	13	**GIRAFFE**	12
9	SHEEP	163	**MONKEY**	13	**BEAR**	11
10	COW	155	**BEAR**	10	GORILLA	11
11	**MOUSE**	152	**BIRD**	10	**MOUSE**	11
12	**RABBIT**	148	**PANDA**	10	**PANDA**	11
13	HIPPOPOTAMUS	143	**RABBIT**	10	**RABBIT**	11
14	**BEAR**	122	RACOON_DOG	8	COW	9
15	RHINOCEROS	116	SHEEP	8	SHEEP	9
16	**BIRD**	115	HAMSTER	7	HIPPOTAMUSE	8
17	**PANDA**	110	LEOPARD	7	HORSE	8
18	CHEETAH	102	RHINOCEROS	7	CHEETA	7
19	SNAKE	102	SPARROW	7	CHIMPANZEE	7
20	ZEBRA	102	ZEBRA	7	RACOON_DOG	7

There are no definite rules for choosing an appropriate number of singular values (dimensions) for the dimensionality reduction (44). Therefore, a six-dimensional solution (6D) was used where the sum of the singular values reached 70% to the entire sum. Accordingly, inter-item cosines were calculated in the 6D space.

### Cosine Profiles

**Figure 2:**
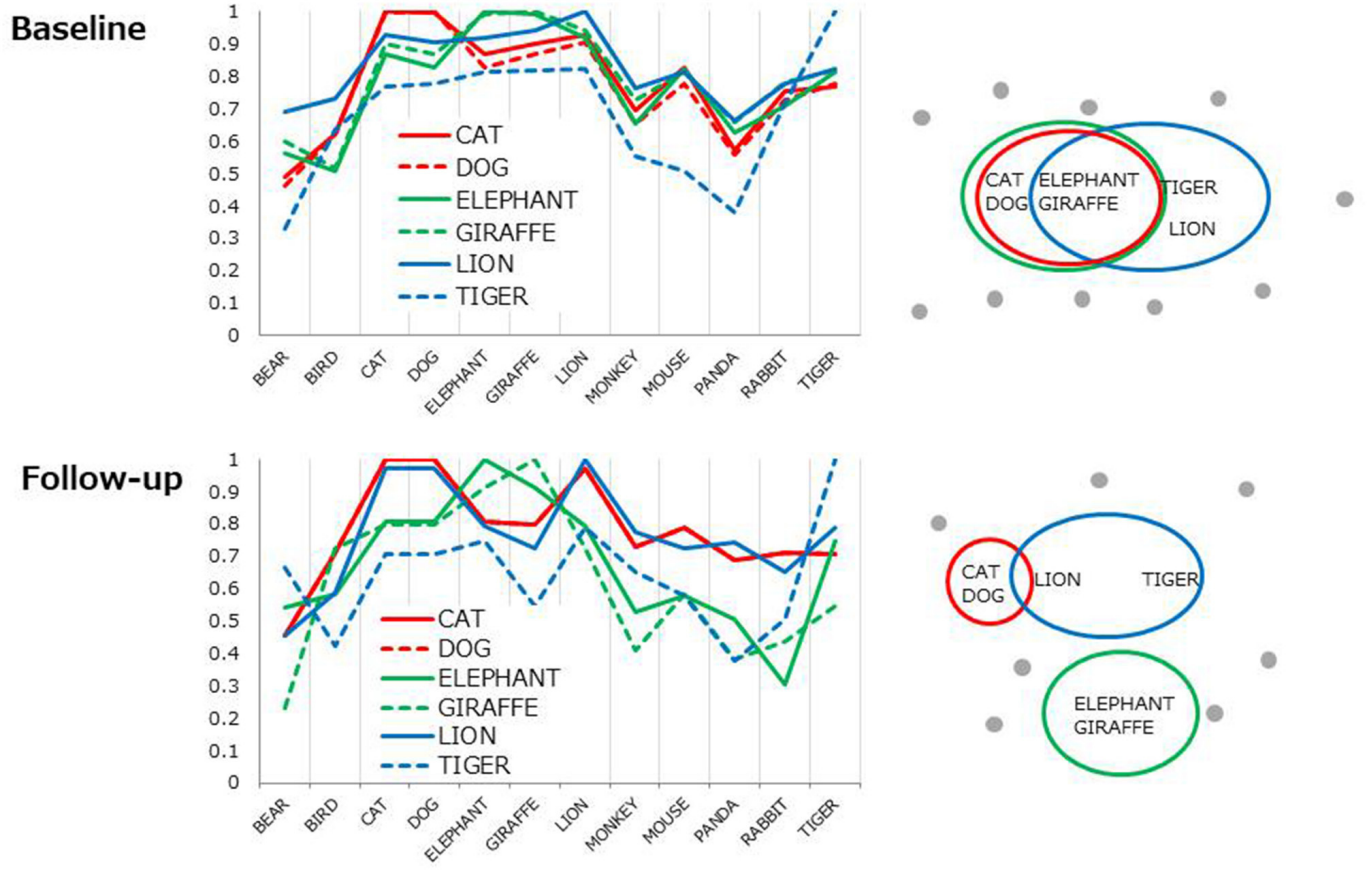
Cosine profiles for the most frequent six items. Each line represents 6D cosine values between one of the six items and the other frequent 12 items. The profile patterns are conceptually illustrated as from of clusters (right).

Each line represents 6D cosine values between one of the top 6 items (e.g., CAT) and the other most frequent 12 items (Table 2, in bold). Overall, cosine values uniformly fluctuated at baseline (Figure 2, top) indicating the lack of distinct clusters (i.e., subcategories). The profiles became more cluster-based at follow-up, yielding a higher cosine within a pair (e.g., CAT-DOG) but lower cosines between pairs (e.g., [CAT-DOG]-[GIRAFFE-ELEPHANT], Figure 2, the bottom) as conceptually shown in Figure 2, right.

### Difference in Magnitude of Correlations

**Figure 3:**
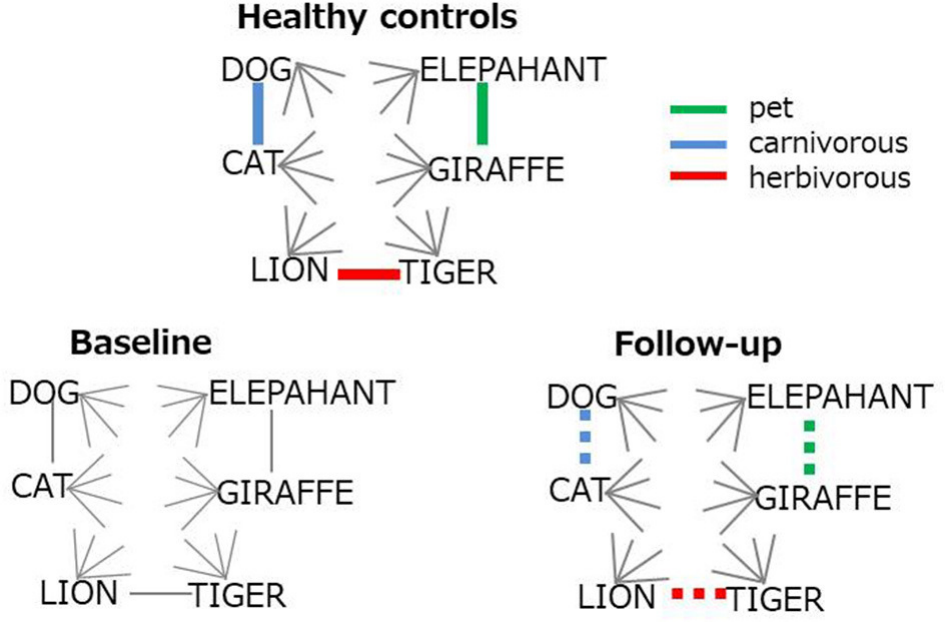
Schematic representation for the improvement on semantic structure. Colored lines represent strong (bold) or restored (dotted) association. Gray lines indicates weak inter-item connection.

The top six items in healthy controls (DOG, CAT, ELEPAHANT, GIRAFFE, LION, and TIGER, Table 2) were used for the comparison between *r*_BH_ and *r*_FH_ to examine how semantic memory in patients became close to that in healthy controls. Table 3 summarizes correlational coefficients and the difference of the magnitude of correlations. The correlation was considerably higher in follow-up (*r*_FH_ = 0.75) than baseline (*r*_BH_ = 0.41), and the difference was significant (*z* = −1.90, *p* = 0.03, 95% CI = −1.06, 0.02). Figure 3 schematically illustrates the cognitive process of the result. For example, LION is more easily and quickly accessed than other items (e.g., ELEPHANT or CAT) when TIGER is recalled.

**Table 3:** Tests for differences in correlational coefficients^a^.

	**SCZ Baseline**	**SCZ Follow-up**	**Healthy controls**
SCZ Baseline	–	0.68	0.41
SCZ Follow-up		–	0.75^*b^
Healthy controls			–

a *Sample size: SCZ = 28; HC = 335*.

b* *r_FH_ = 0.75 > r_BH_ = 0.41, z = −1.90, p = 0.028 (one-tailed), 95% CI = −1.06, 0.02*.

## Discussion

Multi-session tDCS was found to improve semantic memory organization, as evaluated by text-mining analyses of category fluency data, in patients with schizophrenia. The longitudinal comparison of cosine profiles suggests that the semantic association among typical items (animal names) was more cluster-based, as in healthy adults (21) at follow-up compared to baseline (Figure 2). Also, the correlation of cosine values between healthy controls and patients was greater at follow-up than at baseline, indicating that semantic structures of patients approached the normality status after administration of tDCS (Figure 3). Probably, patients at follow-up recalled animal names in a similar manner as did healthy people, referring to subcategory (i.e., pet, carnivorous, herbivorous items, Figure 3) to access items more easily and quickly. Associational memory of this kind would be important in real world settings where meaningful conversations and discourses are taking place. Furthermore, it is possible that impairment of associating information in semantic memory may negatively affect competent linguistic behaviors. In fact, adults who later developed psychosis were found to produce discourses similar to those of children, with presentations of repetitions and a limited scope of vocabulary (45). Likewise, schizophrenia patients with severe formal thought disorder exhibited utterances that are syntactically less complex (e.g., reduction of embedded or dependent clauses) compared to those of first-degree relatives or healthy adults (46). Difficulties in associating information in semantic memory may underlie such restricted linguistic behavior in patients with schizophrenia.

There are several hypotheses to explain deterioration of semantic memory structure in patients with psychiatric conditions [(19), for review]. Some assume structural distortions of memory (47) while others claim poor memory activation (19). In both cases, associational retrieval of stored information would be compromised. Although the current study did not directly address this issue, it is worth pursuing the basis for the impairment to understand higher-order cognition in schizophrenia in further studies.

The number of word outputs itself in the category fluency task was not increased significantly after administration of tDCS (Table 1). This may be partly due to the relatively short duration assessment span (1 month). Possibly, patients tended to repeat a limited variety of items. In fact, type token ratios(TTR), a measure of variety of words, showed only a slight increase in follow-up (baseline: TTR = 0.26, follow-up: TTR = 0.27). Despite, co-occurrences of typical items came to closer to those in healthy adults, as was indicated by the significantly higher correlation in follow-up than baseline (Table 3).

Previous studies support our results with providing the neurophysiological substrate. The left prefrontal region is assumed to be related to the ability of tDCS to improve organizing of information. For example, a previous study (27) found tDCS over the left DLPFC facilitated retrieval of clustered words. A functional imaging study also found that activation in the left frontal region was correlated with categorical clustering in the recall of a verbal learning task (48). These findings are in accord with our result indicating improvement of semantic association in patients with schizophrenia after tDCS treatment over the left prefrontal region.

Although the number of words in the category fluency task was not significantly changed after administration of tDCS, letter fluency was found to be improved in our previous study with the same protocol (25). Meta-analysis results indicate that tDCS over the left ventral inferior frontal gyrus (49) or the left prefrontal cortex (50) increased the number of words produced in the category fluency task.

Results of the current study based on SVD analysis of the category fluency task may add to the usefulness of text-mining analysis in psychiatry, as has been discussed (51–53). Possibly, novel computational linguistic techniques herein reported, i.e., SVD, LSA, and network analysis may contribute to the advance of the National Institute of Mental Health's Research Domain Criteria (RDoC) initiative (54). For example, these techniques may help evaluate the language or declarative memory construct in the RDoC (53).

Several limitations should be mentioned. First, the current study used the data obtained in a previous one-armed open label study (25, 33) that did not adopt sham comparisons. Second, sample size was considerably larger in healthy controls compared to patients. Inequality happened because the former was used as a reference group to estimate normative semantic structure, requiring relatively large sample size. Finally, healthy control subjects were younger, more educated, and in a higher intellectual status compared with patients. However, this demographic bias may not have affected the comparisons of semantic memory structures, because the knowledge about animals is acquired in the early stage of the development (55). Furthermore, the primitive structures, e.g., clustering, are already present in early childhood (56–58); basic semantic structures should be relatively invariant across ages and educational backgrounds.

In conclusion, the current study demonstrated the facilitative effect of tDCS on semantic memory organization in patients with schizophrenia. Semantic associations in these patients approached the normality status after multi-session tDCS. Text-mining analysis was indicated to effectively evaluate semantic memory structures in patients with psychiatric disorders.

## Data Availability Statement

The original contributions presented in the study are included in the article/supplementary material, further inquiries can be directed to the corresponding author/s.

## Ethics Statement

The studies involving human participants were reviewed and approved by Ethical Committee of National Center of Neurology and Psychiatry, Research Ethics Committee of Fukushima University, and Ethical Committee of Osaka University. The patients/participants provided their written informed consent to participate in this study.

## Author Contributions

CS and TS designed the study in collaboration with ZN and RH. ZN, TI, YY, KS, YH, and AS collected and prepared the data. CS conducted the analyses and wrote the initial draft. TS, ZN, and RH critically revised the draft for important intellectual contents. All authors contributed to the manuscript writing.

## Conflict of Interest

The authors declare that the research was conducted in the absence of any commercial or financial relationships that could be construed as a potential conflict of interest.
